# Trophic Strategies of a Non-Native and a Native Amphibian Species in Shared Ponds

**DOI:** 10.1371/journal.pone.0130549

**Published:** 2015-06-23

**Authors:** Olatz San Sebastián, Joan Navarro, Gustavo A. Llorente, Álex Richter-Boix

**Affiliations:** 1 Departament de Biologia Animal (Vertebrats), Facultat de Biologia, Universitat de Barcelona, Avinguda Diagonal, 643, 08028, Barcelona, Spain; 2 CICGE-Centro de Investigação em Ciências Geo-Espaciais, Observatório Astronómico Prof. Manuel de Barros, Alameda do Monte da Virgem, 4430–146, Vila Nova de Gaia, Portugal; 3 Departamento de Herpetología, Aranzadi Zientzia Elkartea-Sociedad de Ciencias Aranzadi, Zorroagagaina, 11, 20014, Donostia-San Sebastián, Spain; 4 Department of Conservation Biology, Estación Biológica de Doñana (EBD-CSIC), Avda. Américo Vespucio s/n, Sevilla, 41092, Spain; 5 Department of Ecology and Genetics, Uppsala Universitet, Norbyvägen 18 D, 752 36, Uppsala, Sweden; Trier University, GERMANY

## Abstract

One of the critical factors for understanding the establishment, success and potential impact on native species of an introduced species is a thorough knowledge of how these species manage trophic resources. Two main trophic strategies for resource acquisition have been described: competition and opportunism. In the present study our objective was to identify the main trophic strategies of the non-native amphibian *Discoglossus pictus* and its potential trophic impact on the native amphibian *Bufo calamita*. We determine whether *D*. *pictus* exploits similar trophic resources to those exploited by the native *B*. *calamita* (competition hypothesis) or alternative resources (opportunistic hypothesis). To this end, we analyzed the stable isotope values of nitrogen and carbon in larvae of both species, in natural ponds and in controlled laboratory conditions. The similarity of the δ^15^N and δ^13^C values in the two species coupled with isotopic signal variation according to pond conditions and niche partitioning when they co-occurred indicated dietary competition. Additionally, the non-native species was located at higher levels of trophic niches than the native species and *B*. *calamita* suffered an increase in its standard ellipse area when it shared ponds with *D*. *pictus*. These results suggest niche displacement of *B*. *calamita* to non-preferred resources and greater competitive capacity of *D*. *pictus* in field conditions. Moreover, *D*. *pictus* showed a broader niche than the native species in all conditions, indicating increased capacity to exploit the diversity of resources; this may indirectly favor its invasiveness. Despite the limitations of this study (derived from potential variability in pond isotopic signals), the results support previous experimental studies. All the studies indicate that *D*. *pictus* competes with *B*. *calamita* for trophic resources with potential negative effects on the fitness of the latter.

## Introduction

One of the critical factors for understanding the establishment, success and potential impact on native species of an introduced species is a thorough knowledge of how these species manage trophic resources [1–3]. Understanding how non-native species are likely to become established in a particular environment is a central challenge in conservation biology. This understanding is relevant to management policies that aim to prevent invasions or eradicate introduced species [4], and to those that aim to diminish the effects of non-native species on native biodiversity, ecosystem processes or community structures [5,6].

There are a number of different ways in which feeding behavior can contribute to the success of an invading species. Overall, two main trophic pathways have been suggested to explain resource acquisition by non-native species: (1) they may exploit novel niche opportunities that most native species are unable to use (opportunism hypothesis), or (2) they may behave aggressively with respect to the resources exploited by natives, displacing them from their preferred niches (competition hypothesis) [7–10]. The study of trophic niche width and resource distribution between species can be a useful tool to determinate trophic patterns and evaluate the effect of non-native species on invaded communities [11–13]. Under the trophic opportunism hypothesis, the presence of non-native species would not be expected to have any effect on the variety of the diet of native species (niche width) [14,15]. In contrast, under the trophic competition hypothesis with the corresponding inter-specific interactions, the niche width of species with less competitive capacity would be expected to increase due to niche displacement to non-preferred resources [16,17].

Temporary ponds and tadpoles are good ecological models for studies of community structure. These ponds are small closed systems with a recurrent annual dry phase. They are critically important habitats for many amphibian species [18,19]. During the desiccation process, resource availability decreases and larva density increases. The quality and quantity of resources influence the capacity of larvae to complete metamorphosis and perform well [20–23], which key in the breeding success of amphibians [24]. Therefore, during pond desiccation, interaction and competition between species increases [25,26] and the introduction of non-native species can influence the community stability of these systems as well as the breeding success of native species [27,28].

In the present study, our objective was to determine the main trophic strategies of the non-native amphibian *Discoglossus pictus* and its potential trophic impact on the native amphibian *Bufo calamita*; the tadpoles of both species mainly inhabit temporary ponds. We aimed to determine whether *D*. *pictus* exploits similar trophic resources as those exploited by the native *B*. *calamita* (competition hypothesis) or alternative resources (opportunistic hypothesis). To this end, we analyzed the stable isotope values of nitrogen and carbon in larvae of both species. This isotope approach has led to great advances in our understanding of trophic ecology, and provides an integrated view of resource consumption, through identifying food strategies and the trophic levels of species (see reviews: [13,29,30].

*D*. *pictus* is one of the few anuran species introduced into Europe over the last century [31,32] and which has expanded along the Mediterranean coast from southeast France into northeast Spain [33,34]. The overlap in the habitat of *D*. *pictus* and *B*. *calamita* tadpoles as well as their similar morphology suggests a potential overlap in their trophic niches [35,36]. Previous laboratory experiments revealed increased competitive capacity of the introduced over the native species [36], but no studies have been conducted in natural conditions to corroborate those results. In agreement with the previous competition experiment, we expect the introduced species to adopt a competitive strategy in co-occurrence with *B*. *calamita* and to displace the native species to alternative and potentially lower-quality resources.

## Materials and Methods

### Ethics Statement

The study zone (Girona) is not a protected area, the present work did not involve endangered or protected species and the work was approved by the competent authorities. *D*. *pictus* and *B*. *calamita* are not listed as threatened by IUCN Red List (http://www.iucnredlist.org). Even so the development of this study has had no impact on the natural populations of two species. Eggs and tadpoles of these species were collected in Girona with support and collecting permits granted Departament de Medi Ambient i Habitatge de la Generalitat de Catalunya (current Departament d'Agricultura, Ramaderia, Pesca, Alimentació i Medi Natural) (SF/272) of the regional authorities of Catalonia. All works was conducted in strict adherence to the Guidelines for the Care and Use of Laboratory Animals at the University of Barcelona and approved by this institution. Procedures had followed the regulations that cover animal housing and experimentation in Catalonia (Spain) contained in Decret 214/1997 of 30th of July and Llei 5/1995 of 21st of June, both from the Generalitat de Catalunya, which apply the European Directive 86/609/CEE to the Spanish law in Catalonia.

### Study Species

The Mediterranean painted frog *D*. *pictus auritus* was accidentally introduced in SE France (Banyuls-sur-Mer) approximately a century ago from Algeria [37]. Today, this species occupies the Mediterranean coast from Montpellier (southeast France) to Vilassar de Mar (Barcelona, northeast Spain), increasing its distribution annually (Information Server Amphibians and Reptiles of Spain-SIARE 2014; http://siare.herpetologica.es/). The natterjack toad *B*. *calamita* is a native species in the invaded area of *D*. *pictus*. Although it has some isolated populations, its distribution range is wide in southwestern and central Europe. In our study area, *D*. *pictus* and *B*. *calamita* mainly breed in ephemeral and temporary ponds. Both species are “opportunistic” breeders, with a reproductive period that starts after periods of rainfall (early spring and early autumn). Due to similarities in breeding strategies and breeding habitat at the larval stage, the two species overlap in time and space during the larval stage [34]. In addition, both species have been described as benthic animals, which live at the bottom of ponds and rasp similar food from submerged vegetation and substrate [35]. In temporary ponds, both species often suffer high mortality related to pond desiccation [38,39]. This process increases competition within and between species for those resources that allow individuals to develop as quickly as possible. There is a relationship between quality and quantity of exploited resources and tadpole growth and developmental rates [20–23].

### Trophic fractionation experiment

To achieve our objective, in addition to sampling and analyzing the isotope values of larvae of *D*. *pictus* and *B*. *calamita* in natural ponds inhabited by both species and in ponds inhabited by only one species, we calculated the isotopic discrimination factors for each amphibian species in a controlled-diet laboratory experiment. The trophic fractionation value is the rate at which animals incorporate the isotope values (Δ^13^C and Δ^15^N) of their diets into their tissues [40,41]. To study the trophic fractionation values of both species, we collected samples of 2–3 clutches for each species from the same study area and transported them to our laboratory at the University of Barcelona (Barcelona, Spain). Egg masses were hatched and the tadpoles were separated in individual plastic containers of 1000 ml with standard dechlorinated water under constant conditions of temperature and photoperiod (12 h dark: 12 h light). During the experiment, the tadpoles were fed with commercial rabbit chow ad libitum (Cuniasa Mater, ASA S.L., Asturias, Spain; 16% protein, 3% lipids, 17% carbohydrate, 10% ash). The temperature during the experiment was maintained between ~20°C, similar to the mean 20–22°C recorded in natural ponds. When the tadpoles reached Gosner stage 39, the same development stage as the tadpoles collected in the field, 21 tadpoles of each species were fasted for 48 hours, euthanized with absolute ethanol and then stored until analysis. To calculate the discrimination factors, we analyzed the stable isotopes in the tadpoles and a subsample of the administrated food. The diet discrimination factors (Δ^13^C and Δ^15^N) for *D*. *pictus* and *B*. *calamita* were calculated as the difference between the isotope ratios of an animal and its diet.

### Trophic niche of both amphibians in free-living conditions

**Fig 1:**
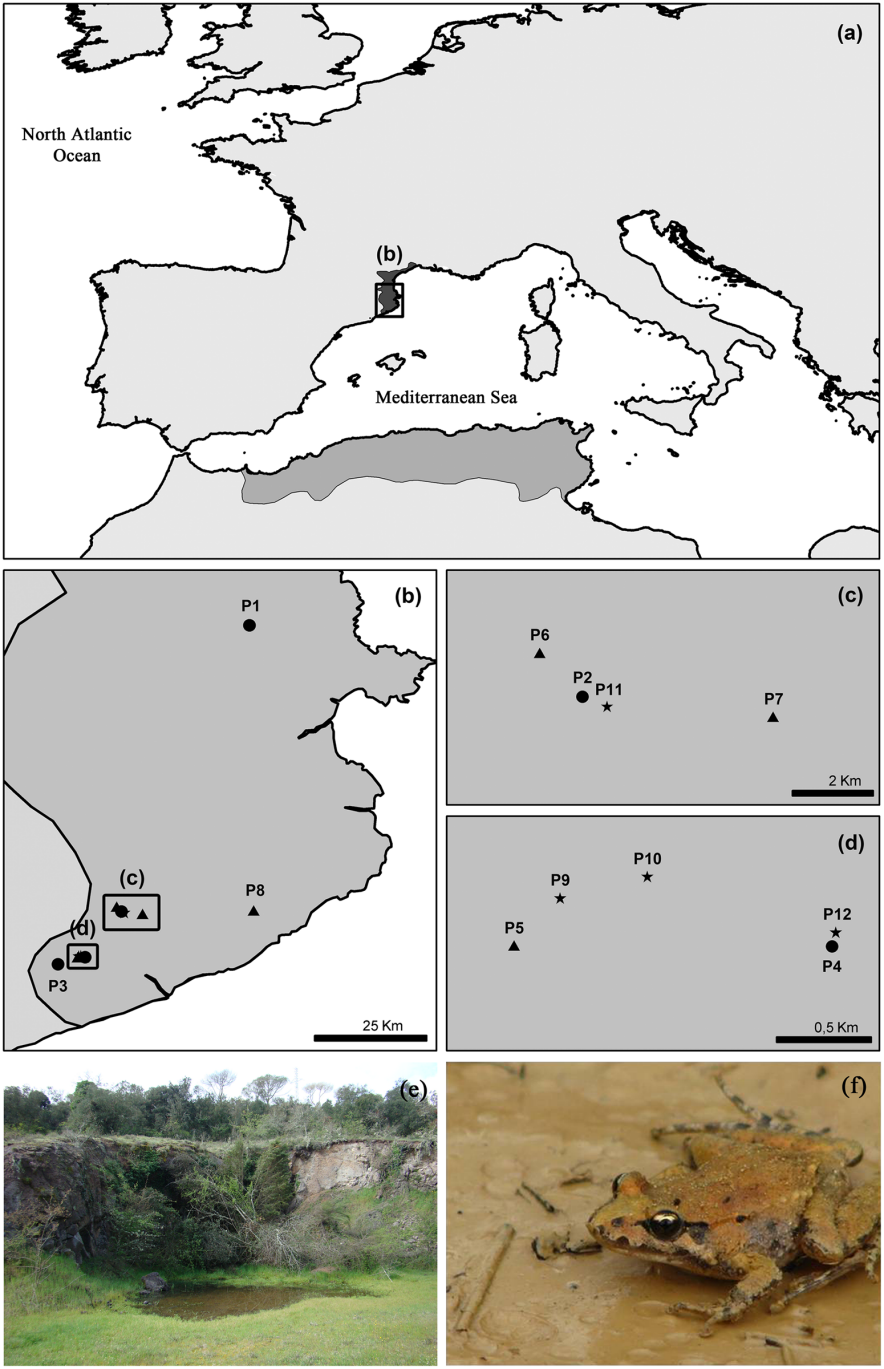
Distribution of *D*. *pictus* and the locations of the study ponds. (a) *D*. *pictus auritus* native (grey) and invaded (black) areas. (b) Study area. (c) and (d) locations of sampled ponds. Dots: *B calamita* non-shared ponds (P1 to P4); triangles: *D*. *pictus* non-shared ponds (P5 to P8); stars: shared ponds (P9 to 12). P1 to P12 correspond to Ponds 1 to 12 in Table 1. (e) Picture of Pond 2. (f) Adult *D*. *pictus*.

Fieldwork procedures were conducted in a natural area situated in northeast Spain (Fig 1). During May 2012 in this study area we monitored 12 temporary ponds with differences for the presence of each species: 4 inhabited only by *B*. *calamita*, 4 only by *D*. *pictus* and 4 inhabited by both species. To reduce the potential isotopic variability associated with contrasting habitats [42], we selected ponds with similar characteristics (Table 1). To reduce the potential effect of the phenology we sampled 10 larvae of each species at a similar developmental stage from each pond (Gosner stage 39; Gosner, 1960). All the tadpoles were held in plastic containers with water and brought to the laboratory.

**Table 1:** Characteristics of selected ponds.

Ponds	Species	Temporality	Temperature	Substrate	Insolation	Area	Depth
		(months)	(°C)	(mm)	(%)	(m^2^)	(cm)
Pond1	*B*. *calamita*	1–2	17.3	<0.6	50–75	6	5–10
Pond2	*B*. *calamita*	1–2	17.7	0.6–2	75–90	26	15–20
Pond3	*B*. *calamita*	1–2	18.5	2–64	>90	9	5–10
Pond4	*B*. *calamita*	3–4	23.4	<0.6	75–90	20	15–20
Pond5	*D*. *pictus*	1–2	22.7	<0.6	>90	8	5–10
Pond6	*D*. *pictus*	1–2	19.6	<0.6	>90	24	15–20
Pond7	*D*. *pictus*	3–4	18.1	<0.6	50–75	8	5–10
Pond8	*D*. *pictus*	1–2	27	<0.6	>90	6	5–10
Pond9	*B*. *calamita* vs. *D*. *pictus*	2–3	30.2	0.6–2	>90	30	5–10
Pond10	*B*. *calamita* vs. *D*. *pictus*	1–2	26.4	<0.6	>90	6	5–10
Pond11	*B*. *calamita* vs. *D*. *pictus*	1–2	15.3	2–64	>90	10	5–10
Pond12	*B*. *calamita* vs. *D*. *pictus*	3–4	23.4	<0.6	75–90	20	10–15

For a quantitative measurement approach of the trophic niche of each species, we analyzed stable isotope values of nitrogen (δ^15^N) and carbon (δ^13^C) of each larva. δ^13^C values provide information on the source of primary carbon and δ^15^N values are related to the trophic level of the organism [43,44]. Stable isotope values reflect the diet over the period during which the tissue analyzed was formed; for tadpoles this is their entire life [45]. To avoid overestimation due to the food remaining in the digestive tract, the tadpoles were held in dechlorinated tap water for 48 hours under fasting conditions; this clears their gut contents. They were then euthanized with absolute ethanol and stored until analysis. We used the same quality and quantity of ethanol for all the samples to avoid any effect on the isotope results.

### Stable isotope analysis

All samples (tadpoles and rabbit food) were dried and homogenized before stable isotope analysis. The homogenization was manual by grinding to a fine powder. As the lipid content of the larvae was low, we did not remove the lipids prior to isotope analysis. Isotope analysis was conducted at the Serveis Científico-Tècnics (University of Barcelona, Spain). Weighed subsamples (between 0.7 and 0.8 mg) of the powered tadpoles and rabbit food were placed in tin capsules. N and C isotope analysis was carried out using an elemental analyzer (Flash EA 1112) coupled to stable isotope ratio mass spectrometry equipment (CF-IRMS). The laboratory uses international standards, which are generally run after every 12 samples: IAEA CH7 (87% C), IAEA CH6 (42% C) and USGS 24 (100% C) for 13C; IAEA N1 and IAEA N2 (21% N) and IAEA NO3 (13.8% N) for 15N. The international standards for N and C are atmospheric nitrogen (AIR) and Pee Dee Belemnite (PDB), respectively. Accuracy was ±0.1% and ±0.2% for δ^13^C and δ^15^N, respectively.

### Statistical analysis

To compare the trophic fractionation values estimated in the controlled-diet experiment between species (*D*. *pictus* and *B*. *calamita*) we used t-tests. As differences in trophic fractionation values between the species were found (see Results section), we corrected the isotopic values of each tadpole collected in the field by the difference in the trophic fractionation estimated with the trophic fractionation experiment between species. After this correction, we tested whether the δ^15^N and δ^13^C values differed between species (*D*. *pictus* and *B*. *calamita*), pond conditions (sharing and non-sharing) and the interaction between species and pond condition, by applying a Generalized Least Squares (GLS) model. Species, pond condition (sharing and non-sharing ponds) and the interaction species-pond condition were included as fixed effects. Considering the non-independence of tadpoles from the same pond, we defined a general correlation matrix assuming that the residuals of the same pond are not independent of each other [46]. Analyses were performed with REML estimation in the *nlme* package using the *corCompSymm* argument in the *gls* function. All statistical analyses were performed in R version 3.0.3.

Trophic niche width was estimated using a Bayesian approach based on multivariate ellipse-based metrics [47]. In particular, we calculated standard ellipse areas (SEAs) for each species in each pond following methods from Jackson et al. (2011) by using the SIAR package [48,49] (http://cran.r-project.org/web/packages/siar/index.html). To detect potential changes in trophic niche width between species and between pond conditions we applied the Mann-Whitney U-test. In addition, in the ponds inhabited by both species we estimated the SEA overlap between the species using SIBER [47].

## Results

### Trophic fractionation experiment

**Fig 2:**
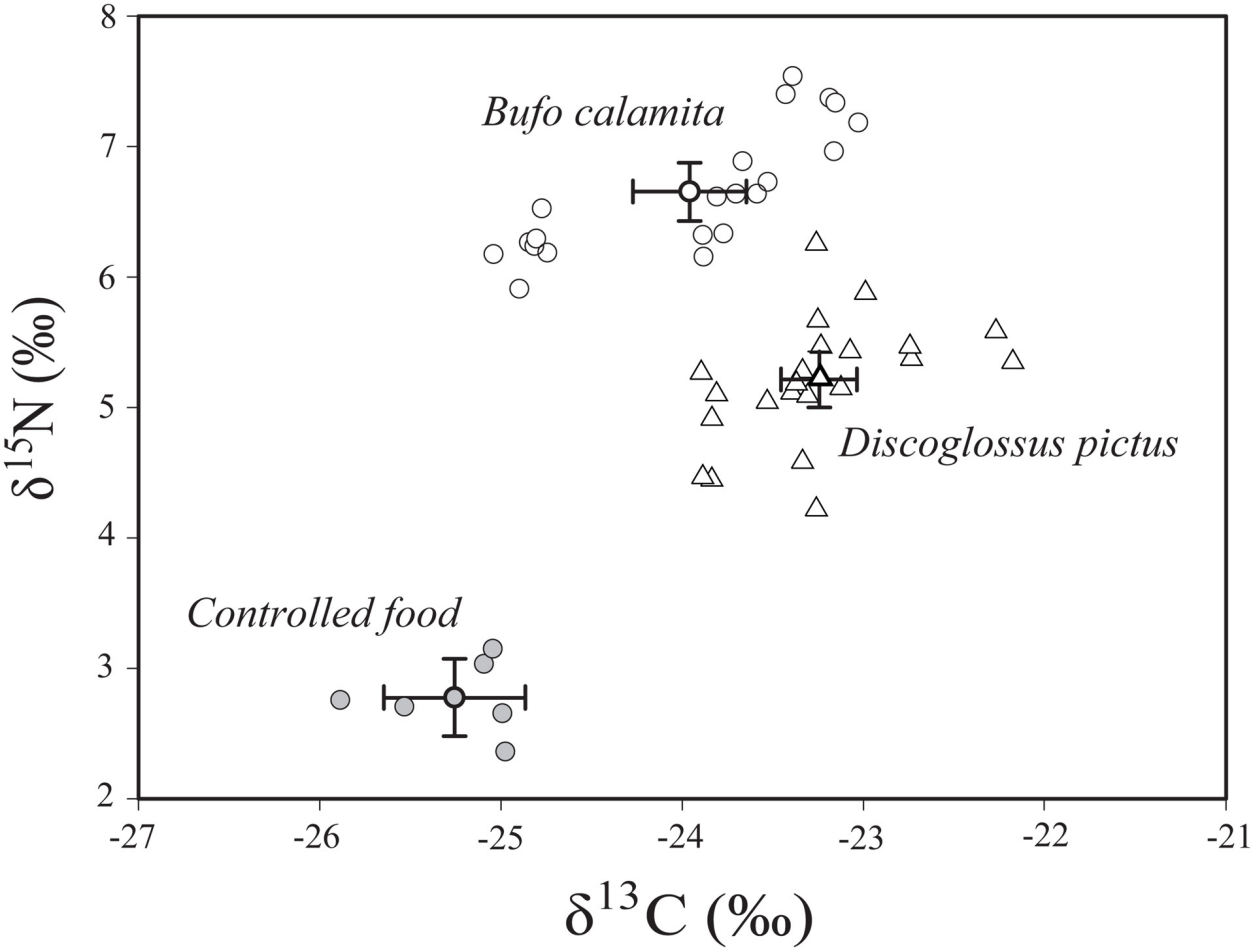
Mean and standard deviation of δ^13^C and δ^15^N values for *B*. *calamita* (filled circles; n = 21), *D*. *pictus* (empty circles) and the controlled diet (filled triangles; n = 21).

We found that both δ^15^N and δ^13^C values differed between *D*. *pictus* and *B*. *calamita* when eating the same food (Table 2). In particular, *B*. *calamita* showed higher δ^15^N and δ^13^C values than *D*. *pictus* (Table 2; Fig 2). Regarding trophic fractionation, *D*. *pictus* showed lower trophic fractionation values for N than *B*. *calamita*, but higher values for C (Table 2).

**Table 2:** Mean ± SD (and range) of δ^15^N and δ^13^C values, and isotopic discrimination factors (Δ^15^N and Δ^13^C) of *D*. *pictus* and *B*. *calamita*.

Species	n	δ^15^N (‰)	Δ^15^N (mean)	δ^13^C (‰)	Δ^13^C (mean)
rabbit food	6	2.77±0.28		-25.25±0.37	
		(0.79)		(0.91)	
*B*. *calamita*	21	6.65±0.48	3.66±0.48	-23.96±0.69	1.56±0.64
		(1.63)		(2.01)	
*D*. *pictus*	21	5.20±0.49	2.23±0.49	-23.25±0.49	0.91±0.49
		(2.05)		(1.72)	
**T-test (between species)**			**t = -3.83** *		**t = 9.62** *

t-test for discrimination factors between species.

*Significant values at p<0.0001.

### Isotope differences among pond typologies

**Fig 3:**
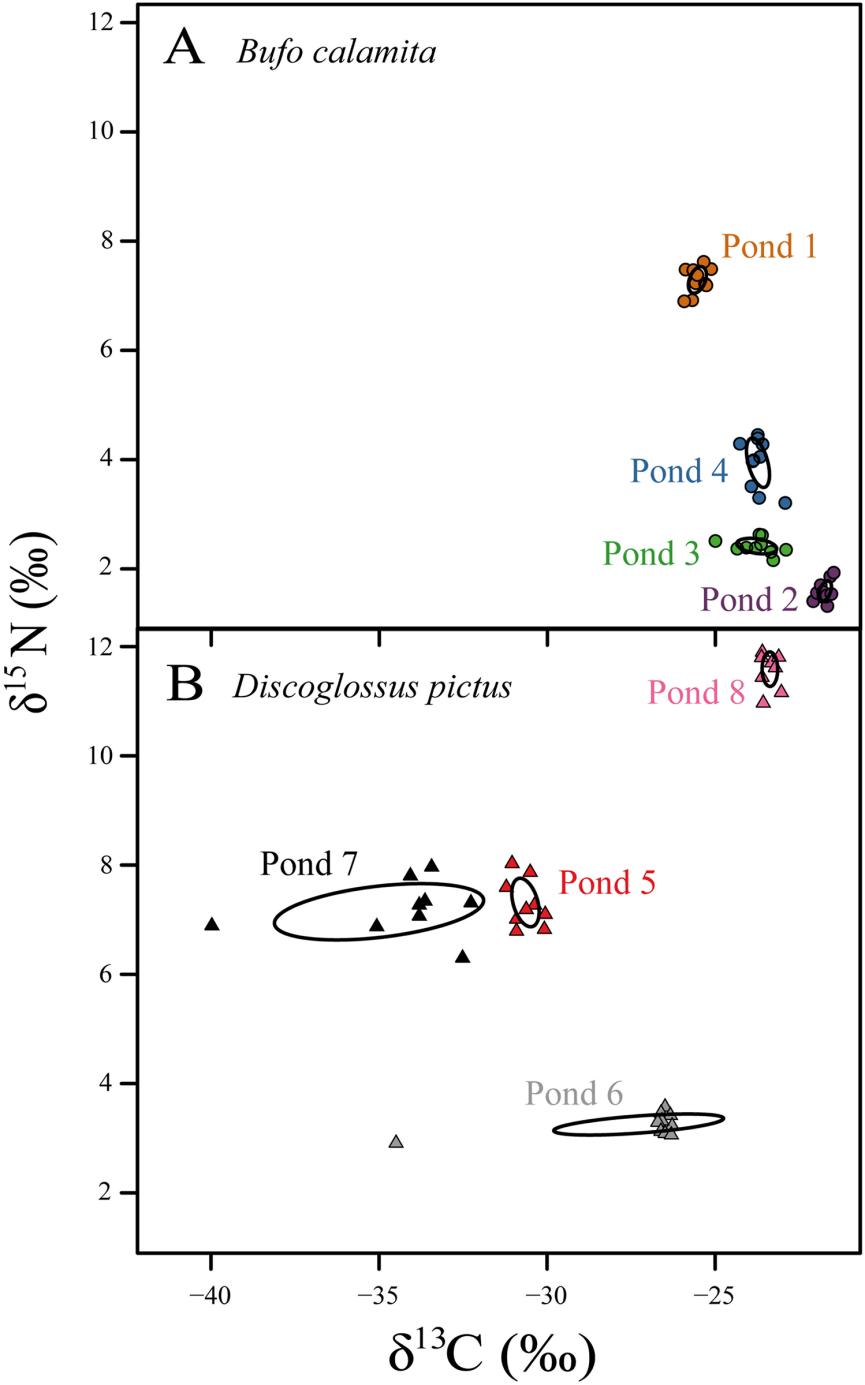
δ^13^C and δ^15^N values and standard ellipse areas for the isotopic niches of *B*. *calamita* (A) and *D*. *pictus (B)* in each pond under non-sharing conditions.

**Fig 4:**
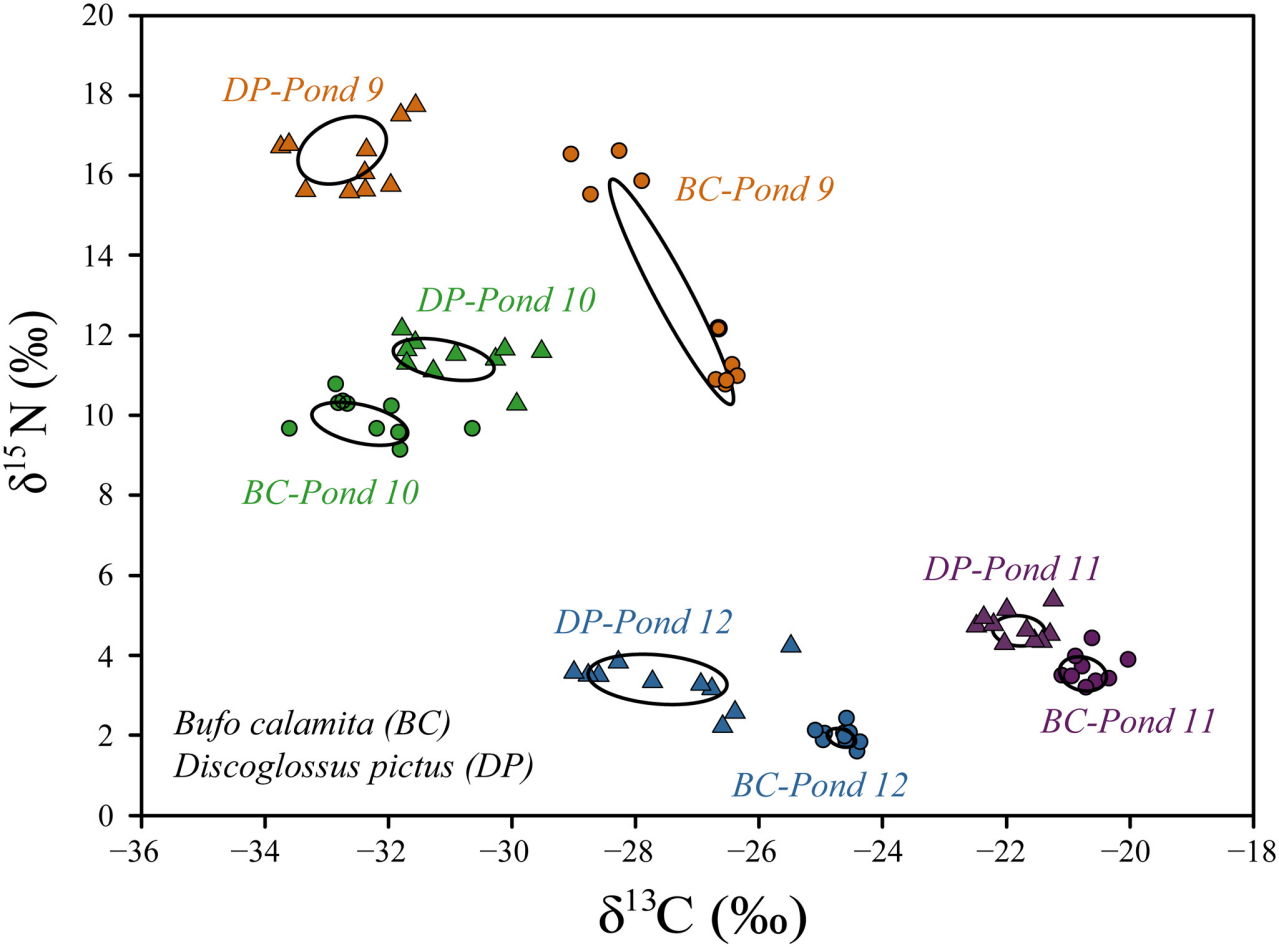
δ^13^C and δ^15^N values and standard ellipse areas for *B*. *calamita* and *D*. *pictus* in the four ponds where the species coexist (A-D).

The δ^15^N values were similar between *D*. *pictus* and *B*. *calamita*. Only pond condition showed a significantly effect on δ^15^N values in both species (Table 3). In particular, in shared ponds, δ^15^N values were higher for both species than in ponds inhabited by only one species (Figs 3 and 4). The δ^13^C values were also similar for the two species, but differed when we compared between shared and non-shared ponds. Specifically, δ^13^C values in share ponds were higher for *D*. *pictus* than *B*. *calamita*. Both species showed a wide range in δ^15^N and δ^13^C values, resulting in range overlap between species in both shared and non-shared ponds (Figs 3 and 4; Table 4).

**Table 3:** Generalized least squares model for effects of the variables species (*D*. *pictus* and *B*. *calamita*), pond condition (sharing or non-sharing), and the interaction between the two on stable isotope values (δ ^15^N and δ ^13^C).

	δ^15^N		δ^13C^	
Variables	F_1, 154_	p	F_1, 154_	p
**Species**	0.18	0.672	0.08	0.781
**Pond condition**	**4.67**	**0.032**	**9.47**	**0.002**
**Species- X Pond condition**	0.16	0.686	0.32	0.569

**Table 4:** δ^15^N and δ^13^C descriptive statistics for two species in shared and non-shared ponds.

Species	Pond condition	Variables	n	Mean (‰)	Minimum (‰)	Maximum (‰)	Std. Dev.
*D*. *pictus*	No-sharing	δ^15^N	38	5.76	1.48	10.46	3.01
*D*. *pictus*	No-sharing	δ^13^C	38	-28.48	-40.69	-22.30	4.78
*D*. *pictus*	Sharing	δ^15^N	40	7.54	0.80	16.30	5.38
*D*. *pictus*	Sharing	δ^13^C	40	-27.48	-33.00	-20.56	4.24
*B*. *calamita*	No-sharing	δ^15^N	40	3.90	1.32	7.62	2.24
*B*. *calamita*	No-sharing	δ^13^C	40	-23.80	-29.36	-21.48	1.66
*B*. *calamita*	Sharing	δ^15^N	40	7.21	1.60	16.62	4.78
*B*. *calamita*	Sharing	δ^13^C	40	-26.26	-33.62	-20.03	4.31

These statistics were evaluated from original isotopic values (without specific diet discrimination correction).

### Differences in the trophic niche width (SEA)

**Fig 5:**
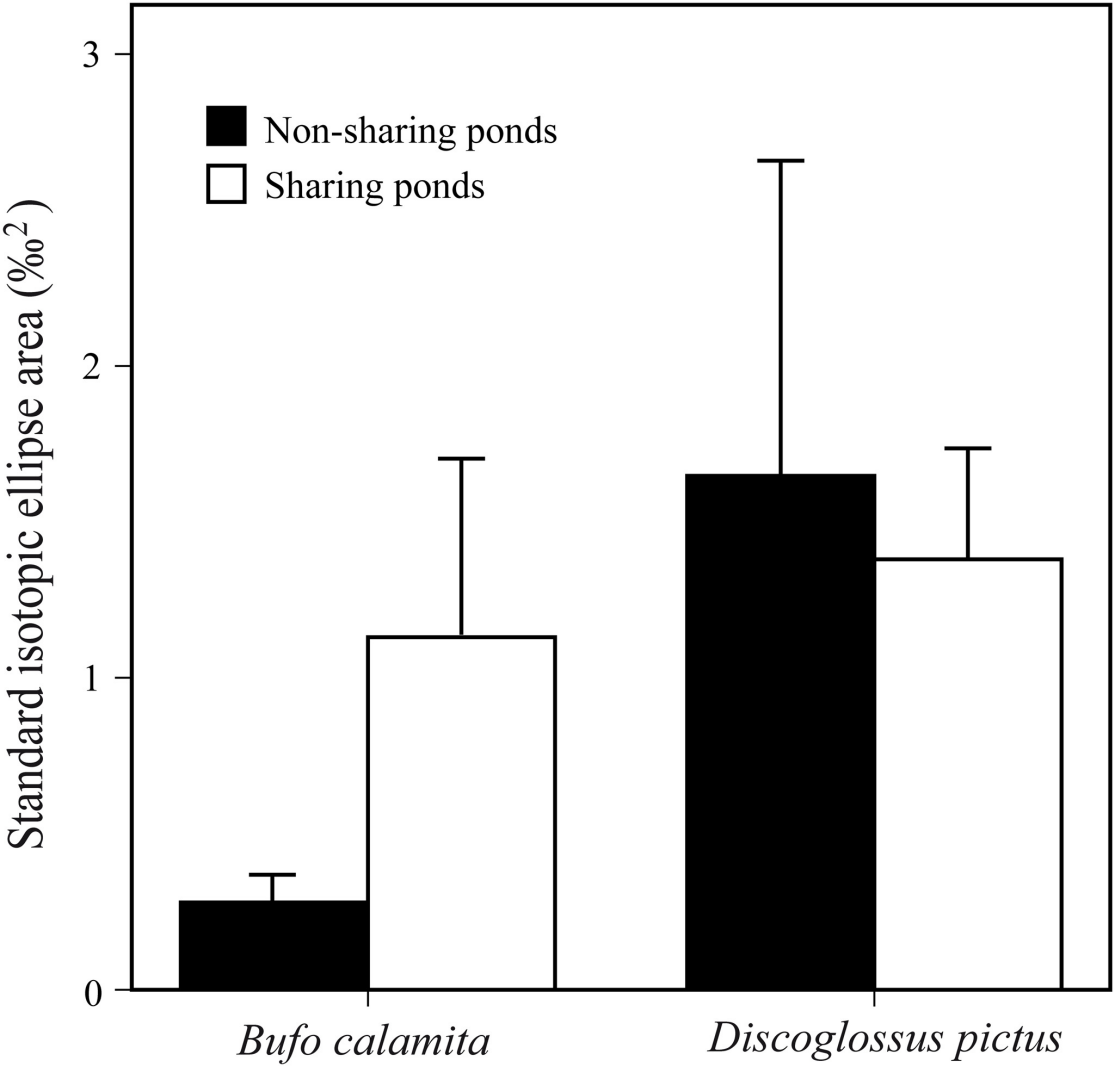
Mean standard isotopic ellipse area for *B*. *calamita* and *D*. *pictus* under sharing and non-sharing niche conditions.

Taking into account both shared and non-shared ponds, *D*. *pictus* showed higher and significantly more marginal SEA values than *B*. *calamita* (Mann Whitney test, U_1,14_ = 14; p = 0.05). *D*. *pictus* registered SEA values of 1.65 and 1.38, in non-shared and shared ponds respectively, while *B*. *calamita* had values of 0.24 and 1.13 in non-shared and shared ponds (Fig 5). We also found a different effect of the pond condition (sharing vs. non-sharing) on the SEAs of the two species. *D*. *pictus* showed no differences in SEA between shared and non-shared ponds (U_1,7_ = 7; p = 0.77). In contrast, *B*. *calamita* showed lower SEAs in non-shared ponds than when it co-occurred with *D*. *pictus* (U_1,7_ = 3, p < 0.001). Moreover, the SEA of *D*. *pictus* and *B*. *calamita* did not spatially overlap when co-occurring (SIBER results always overlap = 0; with an overlap probability of <0.001; Fig 5).

## Discussion

Our study with stable isotopes allowed us to corroborate partially, under field conditions, our hypothesis based on a previous laboratory experiment. We found that tadpoles of both species registered similar δ^15^N and δ^13^C values, which is indicative of similar trophic niches and consequently of potential overlap in resource management between the species. This result is in agreement with previous studies based on *D*. *pictus* and *B*. *calamita* tadpole morphology, which described both as benthic tadpoles that feed by rasping food from submerged areas [35]. Despite their similar diets, *D*. *pictus* and *B*. *calamita* clearly occupy segregated trophic niches when they are present in the same pond. Trophic partitioning is a mechanism that reduces interspecific competition for scarce trophic resources in temporary ponds and thus allows co-existence [50–53]. Niche partitioning has been reported as a type of assembly in both native [54–56] and introduced vs. native communities [57,58]. However, one question we wished to address here is whether *D*. *pictus* adopts a competitive or an opportunistic strategy in this niche segregation.

We found evidence of competition between the introduced and the native species in our study, in consonance with several studies [59–61], which demonstrated that high ecological similarity promotes competition. Competition between non-native and native species can lead to ecological displacement of one species to other resources or even the competitive exclusion of one species [51,62]. These interactions can trigger a variation in stable isotope values due to direct competition for higher quality resources [63,64]. When *D*. *pictus* and *B*. *calamita* co-occurred in the same ponds, the δ^15^N values were higher for both species while the δ^13^C values were only higher for the introduced species. While variation of δ^13^C values suggests different microhabitat exploitation by species within the ponds, the δ^15^N variation may have various interpretations. δ^15^N values indicate the quality of the exploited resources and even the trophic position of organisms [65]. In temporary ponds, space and resources are limited and both interspecific and intraspecific competition for a higher quality diet is unavoidable; this is important for more rapid development and increased fitness [66,67]. The competition for a higher quality diet could increase the isotopic signals for both species. However, the stress derived from competitive interactions could also be the cause of the observed increases in δ^15^N signals. Several studies have shown how various types of nutritional stress (e.g. reduced food intake or starvation) influence the stable isotope signatures of animal tissues by increasing the δ^15^N values [68–70].

The trophic spatial hierarchy may indicate the competitive interaction between the two species or a difference in the exploitation of resources by both. However, the results for SEAs (a proxy for trophic niche width) suggest a displacement of native species and support the hypothesis of competition strategy by invasive species. In all shared ponds, *D*. *pictus* was placed above *B*. *calamita* in the trophic niche representation. Moreover, an increase in the niche width of the native species was found, while *D*. *pictus* maintained the same width. The increment in SEA may be related to searching and the displacement of one species to another type of resource under the presence of a more highly competitive species, when both share diet preferences [71]. The dominant species in general occupies a higher trophic position and displaces the less competitive species to a suboptimal trophic niche [7,9]. Consequently, an increased competitive capacity of the invasive species could lead to the trophic partitioning of the species, displacing the native species to lower quality resources. Previous studies have reported other examples of higher trophic competitive capacity of invasive species in ants [61,72] and fish [73,74].

Amphibians are characterized by their high plasticity and capacity for adaptation to environmental changes. Hence, communities that receive introduced species can probably incorporate shifts in order to minimize potential effects. Niche overlap is often resolved through differences in spatial or temporal niches [75,76], which allow these two species to co-exist. Some authors present the spatial segregation as a possibility of assemblage between *B*. *calamita* and *D*. *galganoi* (a species similar to *D*. *pictus* endemic to the western part of Iberian Peninsula) in species rich-communities [77,78]. On the other hand, *D*. *pictus* and *B*. *calamita* are species with short aquatic developments; therefore, a small displacement in their breeding phenology could be a feasible adaptation among others. The order in which oviposition occurs can have a large effect on the outcome of competitive interactions [79,80]. Especially important are priority effects in coexistence of native and invasive species [81,82]. Be that as it may, the study of the invasive process of *D*. *pictus* offers a good opportunity to gain an understanding of amphibian community assemblage and adaptation to invasions.

### Niche width in invasive species

SEA has been demonstrated to be a useful parameter in the study of invasiveness of introduced species [13,83]. Wide trophic niche has suggested an advantage for invasive species because this trait maximizes the range of resources and prey types that are available to newly settled individuals [84–87]. A narrower niche is thought to be an evolutionary response to an environment that is stable over space and time [88,89]. Our study only compared the niche width of two species (non-native and native) and therefore, we cannot extrapolate to the invasive capacity of *D*. *pictus* or to whether it is a generalist or specialist in terms of its dietary behavior. We could indicate the seemingly greater plasticity of the non-native species studied than that of the native species, but more studies related to the niche width of *D*. *pictus* and its overlap with native species would be required to confirm this. Trophic plasticity joins other plastic traits of *D*. *pictus* already highlighted by other studies that could be the key to its invasive capacity [23,36,90].

### Study limitations and contributions

The study of the trophic niche of amphibians in the field has always had great limitations. Our study is a good example of the utility of stable isotope analysis in this field. Additionally, it provides basic information necessary to development other studies with this technique in two species. Although the technique employed here has been widely applied to other vertebrate groups, such studies are scarce in amphibians and basic information is still deficient [91]. For example, the need to use adequate isotopic fractionation of δ^15^N (denoted Δ^15^N) and δ^13^C (denoted Δ^13^C) in isotope studies is pivotal to a correct interpretation of results. Particularly, the potential differences in the isotopic fractionation between species should be taken into account [40,92]. Although isotopic fractionation values have been calculated for several species from different orders (see review in [40]), very few studies estimate trophic fractionation for tadpoles with values showing differences between species [53,93]. Here, by developing a controlled-diet experiment, we estimated the isotopic fractionation values of N and C for *D*. *pictus* and *B*. *calamita*. The results clearly reveal interspecific differences in isotopic discrimination. These differences could be related directly to variations in the nutritional metabolism of both tadpole species [91], highlighting the importance of using specific factors for each species if we are to obtain correct ecological interpretations of isotope values [94].

Nonetheless, this study has an important limitation. The lack of information regarding resources (availability and isotopic signal) reduces the robustness of our outcomes. Although we chose similar ponds, there could be a certain variability between ponds that may alter the resource values [42]. In the present case we have the advantage of having performed laboratory experiments previously that support our results. Likewise we suggest that the results could be improved by measuring the stable isotope values of the resources and their availability so that isotope mixing models can be applied or our conclusions could be tested by DNA analysis. The confirmation of the competition trophic strategy of *D*. *pictus* in the field is an important concern for amphibian conservation because of the status of this group of vertebrates [95] and to obtain a more accurate view of the effects derived from its introduction. This study is the first evidence of this species' competition ability in the field.

Invasive species can modify or adapt some traits in the course of the invasion process [96]. Some authors have recorded shifts in environmental niche, competition ability or indeed in exploiting trophic resources [97–99]. All shared ponds analyzed in this study are located in areas invaded by *D*. *pictus* 10–20 years ago (SIARE, 2014). Expanding the scope of the study both spatially and numerically would provide an opportunity to test whether our results are general to this non-native species or if it has modified its trophic traits over time. However, the present study is an example of the value of information derived from stable isotopes and its applicability to amphibians. The use of this technique has allowed us to corroborate a previous laboratory hypothesis (the competition strategy by invasive species; [36]). Our results suggest a higher position of invasive species in terms of spatial trophic niche and niche width conservation. Meanwhile, the strategy of *D*. *pictus* and its wide trophic niche strengthen its invasive abilities and have powerful consequences for the fitness of less competitive native species. Currently, studies of competition in amphibian larvae use different approaches, from small laboratory tanks to mesocosms and field enclosures to full ponds (see review in [100]). Even if the use of tanks and other experimental mesocosm approaches have advantages [101], only correlative studies using full ponds and analyzing tadpoles with unrestricted access to the full pond can help us to evaluate the real impact of competition in nature [53,102] and thereby of introduced species on native communities.
